# Evidence for communication of peripheral iron status to cerebrospinal fluid: clinical implications for therapeutic strategy

**DOI:** 10.1186/s12987-020-00190-8

**Published:** 2020-04-16

**Authors:** James R. Connor, Kari Duck, Stephanie Patton, Ian A. Simpson, Lynn Marie Trotti, Richard Allen, Christopher J. Earley, David Rye

**Affiliations:** 1 Department of Neurosurgery, Penn State Hershey Medical Center, University Dr (H110), 850, Hershey, PA 17033 USA; 2 Department of Neural and Behavioral Sciences, Penn State Hershey Medical Center, Hershey, PA USA; 3 Department of Neurology, Emory University, Atlanta, GA USA; 4 Department of Neurology, Johns Hopkins University, Baltimore, MD USA

**Keywords:** Iron, Transferrin, BBB, CSF, Serum ferritin, Restless legs syndrome, Hemoglobin

## Abstract

**Background:**

Iron is crucial for proper functioning of all organs including the brain. Deficiencies and excess of iron are common and contribute to substantial morbidity and mortality. Whereas iron’s involvement in erythropoiesis drives clinical practice, the guidelines informing interventional strategies for iron repletion in neurological disorders are poorly defined. The objective of this study was to determine if peripheral iron status is communicated to the brain.

**Methods:**

We used a bi-chamber cell culture model of the blood–brain-barrier to determine transcytosis of iron delivered by transferrin as a metric of iron transport. In the apical chamber (representative of the blood) we placed transferrin complexed with iron^59^ and in the basal chamber (representative of the brain) we placed human cerebrospinal fluid. Cerebrospinal fluid (CSF) samples (N = 24) were collected via lumbar puncture. The integrity of the tight junctions were monitored throughout the experiments using RITC-Dextran.

**Results:**

We demonstrate that iron transport correlates positively with plasma hemoglobin concentrations but not serum ferritin levels.

**Conclusions:**

The clinical ramifications of these findings are several- fold. They suggest that erythropoietic demands for iron take precedence over brain requirements, and that the metric traditionally considered to be the most specific test reflecting total body iron stores and relied upon to inform treatment decisions–*i.e.,* serum ferritin–may not be the preferred peripheral indicator when attempting to promote brain iron uptake. The future direction of this line of investigation is to identify the factor(s) in the CSF that influence iron transport at the level of the BBB.

## Background

Iron deficiency has been associated with obesity, congestive heart failure, gastrointestinal disorders, and neurological conditions such as restless legs syndrome and attention deficit disorder, whereas iron excess contributes to a number of diseases including neurodegenerative diseases [1–5]. Iron’s contribution to the pathophysiology of these diverse conditions and what place oral and parenteral iron treatments might have in their treatment are not well defined. These voids in knowledge would be advanced by determining whether systemic iron status is communicated to individual end-organs, and if so, what factors mediate this signaling. Pre-anemic iron deficiency and iron deficiency anemia have been recognized as significant global health problems [6] and treatments include oral iron supplements, consumption of iron fortified foods, and the use of intravenous iron for rapid replenishment of iron status. Intravenous iron has been recommended as a treatment for neurological disorders such as restless legs syndrome, and use of iron supplements for children, especially those who have been iron deficient, to promote brain development [7]. We have published the paradigm shifting concept that brain iron uptake is regulated by the brain; challenging conventional wisdom that the blood–brain-barrier (BBB) is a passive conduit for iron transport [8]. It follows then that signals from the extracellular fluid in the brain (including CSF) direct the amount of iron that is to be transported across the BBB. However, do those signals occur independent of peripheral iron status? If so, what might guide the clinical decision to prescribe iron supplements for disorders such as Restless Legs Syndrome (RLS) or for enhancement of brain development and function? The goal of this study was to identify relationships between brain iron transport and the traditional peripheral iron metrics serum ferritin that is reflective of systemic iron status or hemoglobin an indicator of red blood cells and oxygen transport capacity of the blood. Low serum ferritin levels are thought to reflect low body iron stores and low hemoglobin levels indicate anemia [9].

## Methods

We used a dual chamber model of the blood–brain-barrier consisting of bovine brain microvascular endothelial cells grown to confluence on Costar transwell porous filters in the presence of hydrocortisone to induce the formation of tight junctions. The details of this model have been reported previously. [8] Cerebrospinal fluid (CSF) samples (N = 20) collected via lumbar puncture came from an institutional IRB approved biospecimen repository from over 350 individuals being investigated for complaints of excessive daytime sleepiness or central disorder of hypersomnolence at Emory University, and 4 additional samples collected as part of an IRB approved study on restless legs syndrome at Johns Hopkins University. To control for some factors suspected to introduce variance in iron homeostasis, samples were derived solely from females of northern European ancestry between the ages of 35–66 who were who were not considered anemic by consensus standards (i.e., hemoglobin concentrations > 12 g/dL). [10] Samples of CSF and blood were obtained within a range of 24 h of one another (N = 15 of 20), or no more than 3 months apart (N = 5; range 4 days-3 months). Serum ferritin and hemoglobin levels were determined by a standard clinical chemistry laboratory. We divided the cases into equal groups: one having been diagnosed with restless legs syndrome (RLS) versus another having never experienced RLS symptoms or exhibited signs of the disorder (i.e., periodic limb movements of sleep). To investigate the influence of the CSF on iron transport in the BBB model, we placed 600 µl of CSF into the basal chamber and ^59^Fe-AlexaFluor 488-Transferrin and 70 kDa RITC-Dextran into the apical chamber as described previously. Dextran was present to monitor the integrity of the tight junctions throughout the course of the study. [11] The amount RITC-dextran in the basal chamber did not fluctuate by more than 8% over the course of the study which was not statistically significant as determined by *t* test. Samples (50 µl) from the basal chamber were taken at 2-hour intervals over 10 h and this volume (50 µL) was replaced with fresh media at each time point. Only the 10-hour concentrations are reported to reflect the maximum transport. Fluorescence was measured in all samples on a SpectraMax Gemini EM plate reader (Molecular Devices) and ^59^Fe was measured on a Beckman Gamma 4000 (Beckman Coulter).

### Statistics

Prism (GraphPad Software) software was used for all statistical analyses and data graphing. Statistical differences between experimental groups were determined using two-way ANOVA and Bonferroni’s multiple comparisons test. A level of significance of p < 0.05 was used for all differences evaluated. Correlation analyses were performed using the Prism software to determine the linear relationship of iron transport and serum ferritin or serum hemoglobin. Significance was defined by p < 0.05.

## Results and discussion

**Fig. 1:**
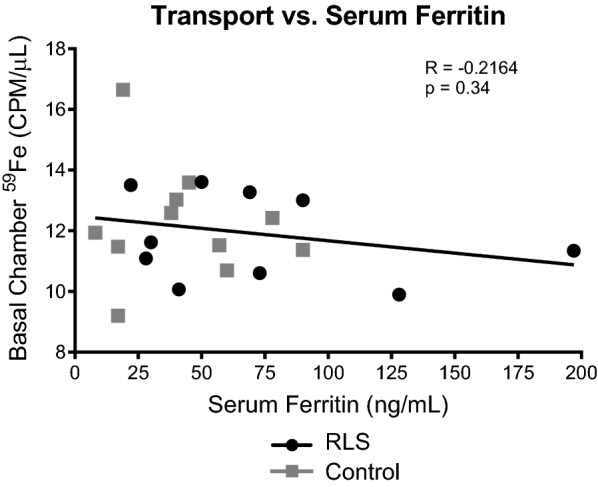
Iron Transport in a BBB model is not correlated to serum ferritin levels. Transport of radiolabeled iron in the bi-chamber model of the BBB was not related to serum ferritin levels. The CSF from patients (RLS or control) was placed into the basal chamber of the model and transferrin conjugated with ^59^Fe was placed into the apical chamber. Measurements were taken from the basal chamber over 10 h. The amount of iron transported at 10-hour time point was plotted against the serum ferritin levels for each patient. Grey squares represent control patients and black circles represent RLS patients

Because the iron content in brain tissue and CSF of RLS patients has been reported to be low, [12, 13] we hypothesized that the CSF of RLS patients might provoke greater transport of iron across our BBB model from the apical chamber (representative of the blood) into the basal chamber housing the CSF. There was, however, no difference by affectation status (i.e., RLS vs. controls) and iron transport. The samples were therefore combined and interrogated for correlations to serum ferritin and plasma hemoglobin concentrations to determine if peripheral iron status could influence transport of iron at the BBB. No correlation between iron transport in the BBB model and serum ferritin was observed (r = − 0.22, p = 0.34; Fig. 1). While serum ferritin is considered the most specific test that correlates with total body iron stores and is as an indicator for iron treatment in RLS [14–16], absence of a relationship between serum ferritin and brain iron uptake in this model suggests that ferritin may not be the preferred biomarker for selecting patients for iron supplementation. This novel finding provides an explanation for the failure of baseline serum ferritin levels in controlled trials to predict clinical efficacy of iron repletion for RLS [17].

**Fig. 2:**
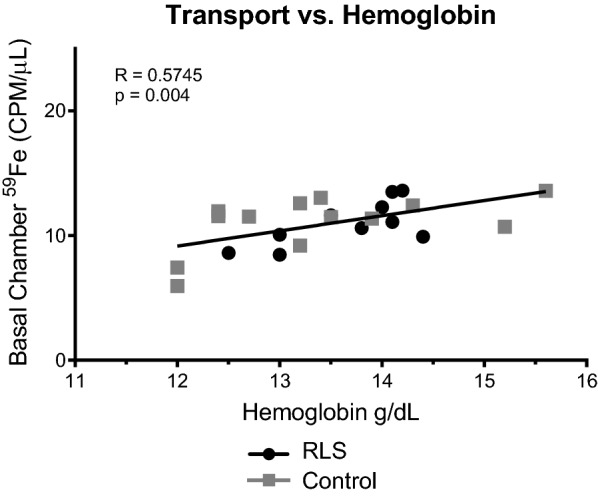
Iron transport in a BBB model is positively correlated with hemoglobin levels. Transport of radiolabeled iron in the bi-chamber model of the BBB was significantly and positively correlated to hemoglobin levels. The CSF from patients (RLS or control) was placed into the basal chamber of the model and transferrin conjugated with ^59^Fe was placed into the apical chamber. Measurements were taken from the basal chamber over 10 h. The amount of iron transported at 10-hour time point was plotted against the hemoglobin concentration for each patient. Grey squares represent control patients and black circles represent RLS patients

We observed a moderately robust positive correlation between iron transport and hemoglobin concentrations (r = 0.57, p = 0.0004; Fig. 2). A key point to emphasize is that despite the common association of hemoglobin with systemic iron levels, the protein’s primary function is in oxygen transport. Our findings are therefore in line with the proposed concept of a hierarchy in which end organs, including the brain, forgo their iron needs to maintain erythropoiesis and the blood’s oxygen carrying capacity [18–21]. For example, in phlebotomized lambs, iron was preferentially directed to red blood cells after liver iron dropped below a critical threshold level, [19] and, the total iron content of red blood cells reportedly increases in tandem with decrements in brain iron [21]. Our data are also consistent with the report that RLS symptoms fail to respond to intravenous iron treatment in individuals with low hemoglobin levels (< 12.5) [22] and suggest in that study, the brain may have participated in an active redirecting iron delivery rather than simply a failure of enough iron to reach the brain in the anemic patients.

It is critical to understand in our experimental design that the endothelial cells in the BBB model were exposed only to CSF in the basal chamber and were not exposed to serum from the patients. Therefore, the oxygen transport capacity of the blood has been communicated to the CSF and this information is, in turn, communicated to the endothelial cells in the BBB model. The result of this communication is that iron is redirected away from the brain when hemoglobin is low, and its uptake into the brain is promoted as hemoglobin increases. In a similar study using the same cell culture model but with CSF from iron deficient or iron sufficient infant monkeys, the correlation between iron release from the endothelial cells and hemoglobin was negative differing from the findings herein. In the study with monkey CSF, we determined iron release from the endothelial cells rather than transport as in the current study therefore the differences in the data likely suggesting release versus transport of iron are regulated differently. Nonetheless, the data from both studies are consistent in revealing communication of the oxygen carrying capacity of blood to the CSF. The factors that participate in this communication are currently under investigation. One factor that communicates between CSF and endothelial cells is transferrin, where we have shown that the iron saturation status of transferrin mediates iron release from endothelial cells in the BBB model [8]. Unfortunately, in these CSF samples we could not distinguish between apo (iron free) and holo (iron saturated) Tf.

Although the clinical implications of this cell culture study should be viewed with caution they are consistent with the lack of serum ferritin status to improve outcome in RLS [17] and the absence of response to RLS symptoms in subjects with lower hemoglobin [22]. These findings provide a number of avenues for further basic science and clinical investigation: (1) hemoglobin levels signal the brain, presumably regarding oxygen availability, which releases some factor(s) into the CSF that alters iron transport across the BBB; (2) efforts to provide iron to the brain could be impacted by hemoglobin levels, even if these are in normal range, (3) the brain is seemingly tolerant of some degree of brain iron deficiency (i.e. in the case of RLS there is hypomyelination and impairments in synaptic dopamine signaling;) but intolerant of diminished oxygen availability (4) the therapeutic implications of the findings in this paper suggest that hemoglobin levels, not serum ferritin should be considered as the peripheral indicator for efforts to replenish brain iron levels.

## Data Availability

The data are available on request in accordance with NIH guidelines. Data sharing is not applicable to this article as no datasets were generated or analyzed during the current study.
